# 2767. Impact of iron limitation on *in vitro* and *in vivo* activity of ceftazidime against *A. baumannii*

**DOI:** 10.1093/ofid/ofad500.2378

**Published:** 2023-11-27

**Authors:** Brianna Eales, James Smith, Cole Hudson, Nazanin Pouya, Vincent Tam

**Affiliations:** University of Houston, Pasadena, Texas; University of Houston, Pasadena, Texas; University of Houston, Pasadena, Texas; University of Houston, Pasadena, Texas; University of Houston, Pasadena, Texas

## Abstract

**Background:**

*Acinetobacter baumannii* (AB) is known to have high rates of multidrug resistance (MDR), new treatment options are critically needed to prevent further spread and mortality. Deferiprone (DFP), a metal chelator, has been shown to induce transient growth inhibition in previous studies, and this could be potentially exploited for therapeutic purposes. The objective of the study was to examine the impact of iron limitation on the *in vitro* and *in vivo* activity of ceftazidime against AB.

**Methods:**

An MDR bloodstream isolate (AB14) recovered from a 50 years-old male patient was used. Ceftazidime (CAZ) minimal inhibitory concentration was determined by microbroth dilution in the presence of DFP. *In vitro* growth profiles were discerned by an automated process (BacterioScan 216Dx) tracking CFU/mL over time. Bacteria were started at a concentration of approximately 5.5 log CFU/mL in tryptic soy broth. The effect of DFP (1000μM) and CAZ (128μg/ml) on bacterial growth was studied either alone or in combination. A neutropenic pneumonia mouse model was used in which the animals were given two doses of cyclophosphamide and one dose of uranyl nitrate intraperitoneally prior to infection. For infection, anesthetized mice were inoculated with approximately 1 × 10^6^ CFU (with and without DFP) via the trachea under laryngoscopic guidance. A humanized regimen consisting of three doses of CAZ over 8h was used to mimic a dose of 2g. Quantitative culture was used to ascertain the bacterial burden of lung tissue immediately and 8h post infection.

**Results:**

MIC of CAZ was reduced from > 256μg/mL to 64, 32, and 1μg/mL in the presence of 500, 1000, and 1500μM of DFP respectively. A combination of subinhibitory concentrations of CAZ and DFP resulted in minimal (< 0.5 log CFU/mL) growth in 20h. The lung bacterial burden of the control groups increased ≥ 1 log CFU/g after 8h, whereas the combination group (CAZ + DFP) stayed comparable to baseline. Control groups compared to combination was statistically different p< 0.05).

**Conclusion:**

These results support that limiting iron has the potential to be a novel approach to treating MDR AB infections. Further research is warranted to broaden the scope to encompass additional clinical isolates, and to optimize the approach as a therapeutic solution.

**Disclosures:**

**All Authors**: No reported disclosures

